# Collaborative development of course feedback with students for psyched up. Put more in, get more out

**DOI:** 10.1192/j.eurpsy.2021.1581

**Published:** 2021-08-13

**Authors:** L. Zac-Williams, S. Kohara, A. Canon, C. Saunders, S. Zhang

**Affiliations:** 1 Gkt School Of Medical Education, King’s College London, London, United Kingdom; 2 Lambeth Child And Adolescent Mental Health Services, South London and Maudsley NHS Foundation Trust, London, United Kingdom; 3 Lishman Unit, Bethlem Royal Hospital, South London and Maudsley NHS Foundation Trust, London, United Kingdom; 4 Service For Complex Autism And Associated Neurodevelopmental Disorders, South London and Maudsley NHS Foundation Trust, London, United Kingdom

**Keywords:** Evaluation, Medical Education, Collaboration

## Abstract

**Introduction:**

PsychED Up is an extra-curricular course for 3^rd^ year medical students at King’s College London delivered by psychiatry trainees, senior students and actors. It focuses on the hidden medical curriculum, exploration of holistic care and communication skills.

**Objectives:**

Develop a responsive and sustainable template for course evaluation Obtain rich and specific feedback across multiple domains which can be translated into course improvements Work collaboratively with former students Empower current students with the knowledge that their input is valuable

**Methods:**

Embedded evaluation in start of term course development sessions to engage faculty in evaluation processes Faculty survey to determine what student feedback would be most useful Questionnaire finalised Collaborative design and refinement of questions, confirmed sub-sections and scope

**Results:**

Revised questionnaire: - Included rationale at the start - Questions tailored to faculty development needs - Greater quantity of prompted questions - Specific questions for large group presentation, small group teaching, actors’ performances and students’ reflections - Quantitative ratings and open-space questions thoughtfully paired Reduced time between sessions and obtainment of feedback Quality and quantity of feedback: - High response rates: 32/30 (2 duplicates) mid-term, 29/30 end-of-term - High-quality filling of open-space feedback allowed consolidation of themes to improve the course

**Conclusions:**

Co-designed questionnaire brought focus and organisation to questions leading to richer, more personalised responses for faculty More detailed reflections were attributed to better student understanding of the questionnaire rationale, and knowledge that they would aid course improvement Created a robust system for collecting long-term feedback for PsychED Up and will continue making iterative amendments

